# Stramonium in Neuralgia

**Published:** 1860-10

**Authors:** A. Young

**Affiliations:** Prescott, Wis.


### STRAMONIUM IN NEURALGIA.

By A. YOUNG, M. D., of Prescott, Wis.

*Messrs. Editors*,—Permit me to call the attention of your readers, to the value of Stramonium in Neuralgia.

I am aware that this narcotic is sometimes administered in this intractable disease, but so far as my own observation extends, it is by no means a common remedy. Here in the West where the Intermittent form of Neuralgia is so prevalent, Quinine and Carb. Iron are principally relied upon in its treatment, yet these not unfrequently fail to arrest it. I have, however, to meet with the first instance that has failed to yield to Stramonium. In some of the cases in which I have used it, Quinine, Carb. Iron, Opium, Aconite, Chloroform, had been tried without success.

Although I have used it principally in Intermittent Neuralgia, I have also found it superior to any other remedy in that department upon Spinal irritation, or connected with general Hyperæsthesis in females—Pleurodynia, &c.

The mode in which I have given it in the intermittent form, is gr. j. of Tilden's Ext. Stramon. Fol. every two or three hours during the intermission, until the system is decidedly affected, indicated by dilated pupil, disordered vision, vertigo, and often hallucinations or mild delirium. When given to this extent, it will generally be found unnecessary to repeat it. Anything less than this will be of comparatively little value. In the other forms of Neuralgia, it is not usually necessary to push the remedy so far.

Beyond the temporary effects following its administration, I have never seen the slightest inconvenience result from its use.

Subsequent constitutional treatment is of course often demanded for the relief of debility, &c.

